# Optimizing water use efficiency and fruit quality of watermelon under mulched drip irrigation in arid regions

**DOI:** 10.3389/fpls.2025.1662575

**Published:** 2025-11-24

**Authors:** Zhiyuan Liu, Hengjia Zhang, Shouchao Yu, Zeyi Wang, Chenli Zhou, Haiyan Li

**Affiliations:** 1 College of Agriculture and Biology, Liaocheng University, Liaocheng, China; 2 College of Water Conservancy and Hydropower Engineering, Gansu Agricultural University, Lanzhou, China

**Keywords:** deficit irrigation, drought, fuzzy matter-element, photosynthesis, water use efficiency, fruit quality

## Abstract

**Introduction:**

The Hexi Oasis is located in the arid region of northwest China and is a crucial area for agricultural development. The region has a dry climate with scarce rainfall and a severe shortage of water resources. It has long relied on the traditional flood irrigation method, which has led to low efficiency in the utilization of water and soil resources and has hindered the sustainable development of agriculture.

**Methods:**

This research was conducted in the Hexi Oasis from 2020 to 2021 using "New Farmer 8" watermelon as the experimental material. A field experiment was carried out to systematically explore the comprehensive effects of different water deficit patterns during different growth stages on the photosynthetic characteristics, yield, quality and water use efficiency of watermelons. Five treatments were set up: T1 with mild water deficit at both the seedling and mature stages (60%–70% FC, FC being the field capacity), T2 with mild water deficit at the seedling stage and moderate water deficit at the mature stage (50%–60% FC), T3 with moderate water deficit at the seedling stage and mild water deficit at the mature stage, T4 with moderate water deficit at both the seedling and mature stages, and CK with full water supply throughout the growth period (70%–80% FC) as the control.

**Results:**

The responses of watermelon photosynthesis, yield, and quality to different water deficit patterns were compared and analyzed to provide a scientific basis for efficient watermelon cultivation in arid oasis areas. The results showed that water deficit significantly reduced the net photosynthetic rate (*Pn*), transpiration rate (*Tr*), and stomatal conductance (*Gs*) of leaves, and the reduction increased with the severity of water deficit, with the reduction in *Pn* being smaller than that of *Tr *and *Gs*. Compared with CK, all water deficit treatments increased the irrigation water use efficiency (IWUE), with the highest IWUE in the T2 treatment (0.78 t·ha^-1^·mm^-1^), which was significantly higher by 10.50% than that of CK. The yield of the T1 treatment was the highest and showed no significant difference from CK, followed by T2, with no significant difference between T1 and T2. Water deficit treatments significantly increased the contents of soluble solids (SS), soluble sugar (SU), vitamin C (Vc), and soluble protein (SP), with the largest increase observed in the T2 treatment. The entropy weight–fuzzy matter-element comprehensive evaluation showed that the T2 treatment had the highest comprehensive score, followed by the T1 treatment. In the arid oasis area of Hexi, the multi-stage irrigation pattern of mild water deficit at the seedling stage combined with moderate or mild water deficit at the mature stage (T2 or T1) could significantly improve water use efficiency and fruit quality while maintaining yield, which represents a feasible strategy for achieving water-saving and high-quality watermelon production.

## 1 Introduction

The Hexi Oasis, located mainly in the arid region of northwest China, is a critical integrated agricultural commodity food production base in Gansu Province (Zhao et al., 2022). However, this region has an arid climate, scarce precipitation, and water resources, and agricultural production is still commonly managed by a traditional extensive irrigation regime, which results in low productivity of farmland and water resources (Wang et al., 2019; Ablimit et al., 2022). Recently, ecological degradation problems have become more and more serious, such as over-expansion of the oasis scale, groundwater pollution, and over-extraction, which seriously restrict the sustainable development of the local ecosystem and specialty melon industry (Wang et al., 2020c; Zhao et al., 2023). Furthermore, rising global temperatures intensify drought severity, threatening agricultural water security (Franco-Navarro et al., 2025). Severe drought stress inhibits crop growth and significantly reduces yields. These losses not only jeopardize regional food security but also underscore the global challenge of increasing food production amid increasingly scarce water resources (Valenzuela and Anderson, 2011). Therefore, it is urgent to develop water-saving agriculture to achieve rational and efficient utilization of limited water resources, thereby promoting the sustainable development of arid oasis areas.

Deficit irrigation achieves water saving, yield stabilization, and quality regulation by applying a certain degree of water stress to certain growth stages of the crop, using the crop’s adaptations (Fereres and Soriano, 2007; Chai et al., 2016). A study showed that water deficit (WD) treatments significantly saved irrigation, improved watermelon fruit quality, and increased water productivity compared with adequate irrigation (Kuscu et al., 2015). Among others, rootstock grafting under WD conditions increased watermelon fruit number, yield, water productivity, and root volume and surface area (Yavuz et al., 2020). Humus inputs under deficit irrigation raised watermelon yield and water use efficiency (WUE), and significantly improved soil quality and saved irrigation (Qin and Leskovar, 2020). However, the WUE increased with increasing fertilizer application at the same level of irrigation and the combined benefits of watermelon were better in the moderate water fertilizer treatment (Jin et al., 2021; Zhang et al., 2021). At the same level of fertilizer application, controlling alternative split-root drip irrigation increased photosynthetic rate, plant growth, and NPK uptake by 10.1%, 25.5%, and 29.1%, respectively, compared with conventional drip irrigation over the entire growing season (Wang et al., 2022). As can be seen, current studies on WD in watermelon are mainly focused on irrigation, cultivation methods, and nutrient inputs. Additionally, the under-film drip irrigation technique integrates the advantages of both drip irrigation technique and mulch cultivation technique (Karlberg et al., 2007; Dong et al., 2018; Liu et al., 2020), which provides a new way to develop efficient water-saving irrigation techniques in inland arid areas and has been vigorously promoted and widely applied in the northwestern arid regions of China (Liu et al., 2012). Thus, under-film drip irrigation coupled with deficit irrigation can further reduce water losses from farmland and improve water productivity.

Watermelon (*Citrullus lanatus* L.) is a sprawling annual vine belonging to the Cucurbitaceae family. Its fruit is thirst-quenching, nutrient-rich, and used as medicine, and occupies an important position in the world’s horticultural industry. According to the FAO, 2021 statistics, the global harvest area and yield of watermelon is 3.03×10^6^ ha and 101.56×10^6^ t respectively, of which China accounts for 46.53% and 59.81% respectively (FAO, 2021). The distinctive light and heat climate of the Hexi Oasis is well suited to the growth of watermelon and melon crops. Yet watermelon has a high water consumption and is sensitive to changes in soil moisture, especially during its critical water demand period, when increases or decreases in the water supply can significantly affect the growth, fruit yield, and quality (Orta et al., 2003; Rouphael et al., 2008; Özmen et al., 2015; Wang et al., 2020b). Hence, the rational allocation of regional water and soil resources plays a crucial role in enhancing water use efficiency in arid regions, optimizing crop quality, and promoting the health of farmland ecosystems.

Although there has been more investigation on watermelon deficit irrigation to date, it has mostly focused on grafting cultivation. The abundance of light and heat resources and large diurnal temperature differences in Hexi oasis region of China has enabled the rapid development of the oasis watermelon industry (Zhang and Liao, 2019). However, watermelon production in this region faces the situation of unstable yield and low quality and water utilization. At the same time, there is a paucity of comprehensive studies on watermelon physiological parameters, yield, and quality with deficit irrigation techniques, especially few studies have been reported about the desert oasis area. Thus, it is necessary to investigate the efficient water-saving cultivation mode of watermelon in the Hexi desert oasis area. In recent years, mathematical models such as principal component analysis (PCA), grey relational analysis (GRA), and the technique for order preference by similarity to an ideal solution (TOPSIS) have been mainly used in agriculture to comprehensively evaluate multiple indicators including fruit yield and water use efficiency. Due to the possible differences in results obtained from different models, it is necessary to introduce more extensive and reliable models and data to optimize the water management strategies for crops. The fuzzy matter-element model has been proven to be applicable to multi-objective comprehensive optimization problems, but its application in the agricultural field is still rare. This paper combines the entropy weight method with the fuzzy matter-element model and applies it to the comprehensive evaluation of water deficit irrigation patterns for watermelons. This method can objectively balance the weights of multiple indicators such as yield, quality, and water use efficiency, and quantify the proximity of each treatment scheme to the ideal scheme, thereby overcoming the limitations of traditional methods where the determination of weights is highly subjective or only focuses on a single optimization objective. Accordingly, we took oasis watermelon as the study object. We tested the hypothesis that there may be a significant improvement of water relation parameters like water use efficiency and fruit quality when multi-stage water deficit is applied with under-mulched drip irrigation in the oasis region. The main objective was to investigate: (1) the effect of multi-stage WD mode on photosynthesis, yield, quality, and IWUE of field watermelon under film drip irrigation conditions, and (2) how to decide the deficit irrigation mode based on yield, quality and water compromise using the weighted fuzzy matter-element model, which would provide theoretical basis and technical support for water-saving, yield-increasing, high-efficiency and green production of oasis watermelon in cold and arid environment.

## 2 Materials and methods

### 2.1 Description of the study site

**Figure 1:**
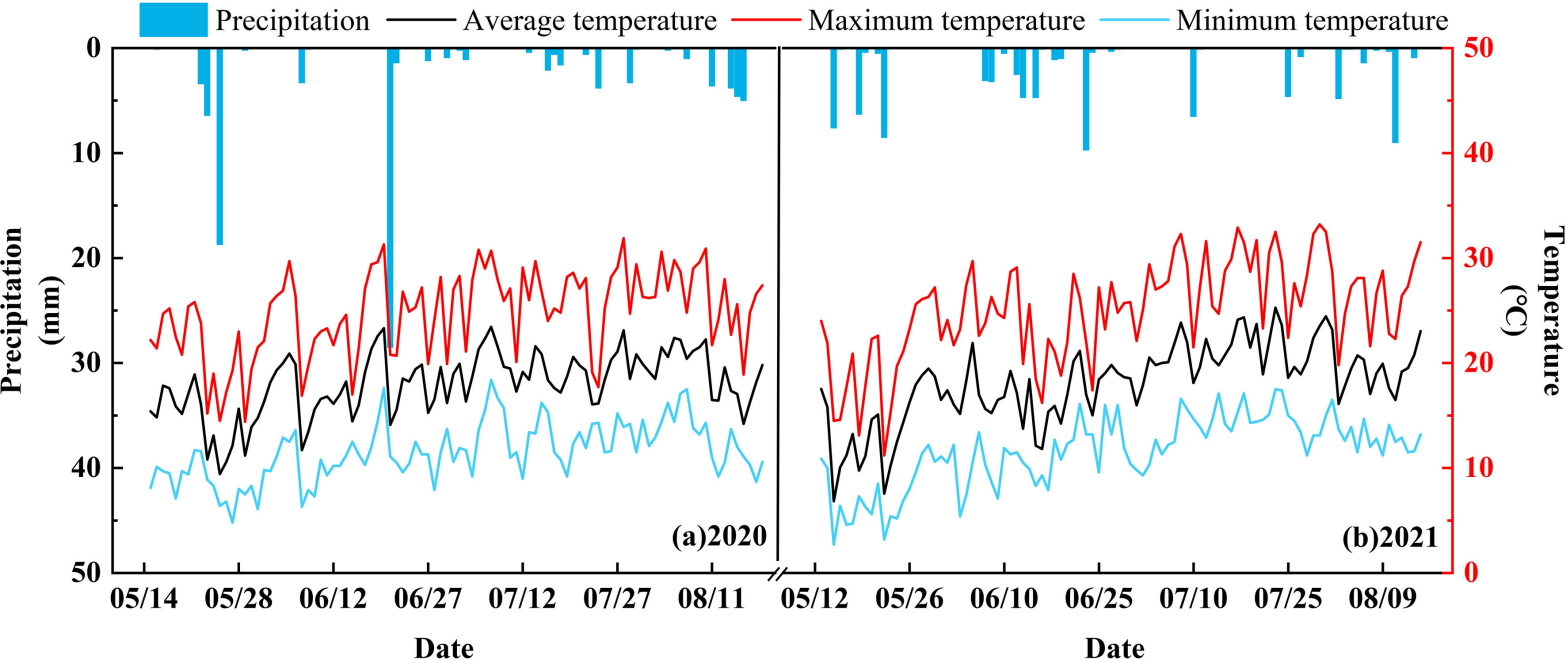
The precipitation, daily maximum temperature, daily average temperature, and daily minimum temperature data for Sanbao Town, Minle County, in 2020 and 2021.

This study was conducted at the Yimin Irrigation Experiment Station from May to August 2020 and 2021. The site is located in Sanbao Town, Minle County, Gansu Province (100°43′E, 38°39′N, altitude 1,970 m). The trial region is dry with little rain (multi-year average of only about 200 mm), with high evaporation (multi-year average of 2,000 mm), long sunshine hours (multi-year average of 3,000 h), an annual frost-free period of 118 d, large diurnal temperature differences, and insufficient water sources. The total precipitation during watermelon fertility in 2020 and 2021 was 176 mm and 102.5 mm (**Figure 1**), respectively, and the effective precipitation (single precipitation > 5 mm) was 68.00 mm and 61.40 mm, respectively, both concentrated in July and August. The soil in the test site is light loam. The maximum field capacity (FC) is 24%. The soil volume mass is 1.46 g·cm^-3^. The pH value is 7.2. The organic matter, alkali-hydrolyzed nitrogen, available phosphorus, and available potassium of the 0–20 cm tillage layer is 12.8 g·kg^-1^, 63.5 mg·kg^-1^, 13.1 mg·kg^-1^, and 192.7 mg·kg^-1^, respectively. The groundwater level is low and there is no salinization.

### 2.2 Experimental design

**Figure 2:**
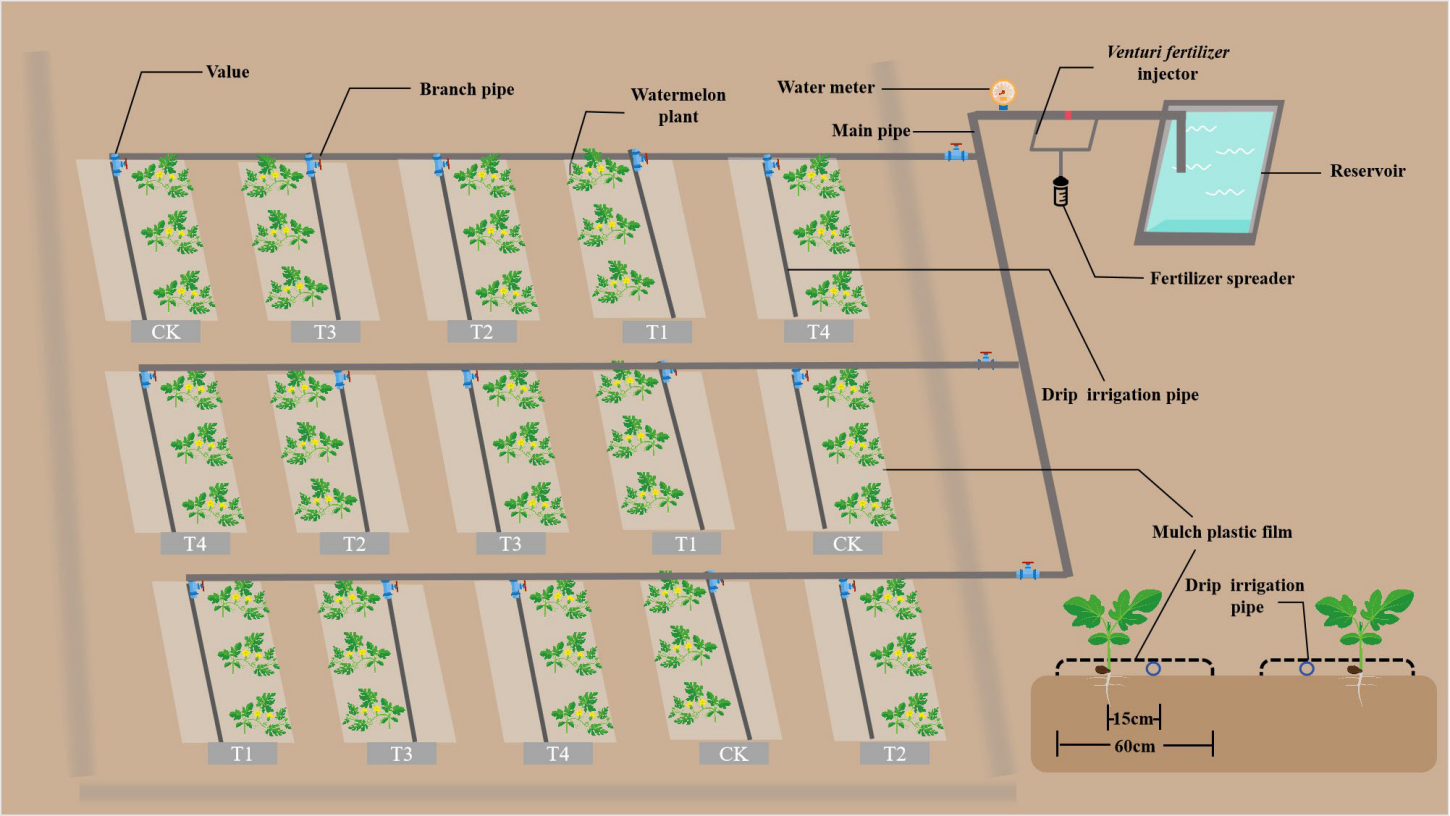
Schematic diagram of irrigation system and planting pattern for watermelon.

The watermelon reproductive period was divided into five stages: seedling stage, vine stage, flowering and fruiting stage, expansion stage, and maturity stage. As the trial site is located in an inland arid zone, considering the regional production practice, a field capacity (FC) of 70–80% was set as adequate water supply, 60–70% FC as a mild deficit, and 50–60% FC as a moderate deficit, and the upper and lower water limits were designed to be in line with the regional reality. Considering that the water demand of watermelon is at its peak during the vine stage and expansion stage, and the plants are affected by water deficit, four treatments (T1–T4) and one control (CK) was set up in the experiment, namely T1 with mild (60–70% FC) water deficit at both seedling and maturity stage, T2 with mild (60–70% FC) water deficit at seedling stage and moderate (50–60% FC) water deficit at maturity stage, T3 with moderate (50–60% FC) water deficit at seedling stage and mild (60–70% FC) water deficit at maturity stage, T4 with moderate (50–60% FC) water deficit at both seedling and maturity stage and CK with adequate (70–80% FC) water supply throughout the reproductive period (**Table 1**). The irrigation method was drip irrigation with branch ball valves and water meters in each plot unit. The trial was laid out in single-factor randomized groups, replicated three times, with a plot size of 7 m × 2.4 m and plant spacing of 30–35 cm (**Figure 2**). The watermelon variety tested was ‘Xinong 8’, sown on 30 April and harvested on 19 August 2020, and sown on 1 May and harvested on 15 August 2021.

**Table 1:** Experimental design.

Treatment	Water deficit control (lower limit to upper limit, % FC)				
	Seeding	Vine	Flowering and fruiting	Expanding	Maturity
T1	60%-70%	70%-80%	70%-80%	70%-80%	60%-70%
T2	60%-70%	70%-80%	70%-80%	70%-80%	50%-60%
T3	50%-60%	70%-80%	70%-80%	70%-80%	60%-70%
T4	50%-60%	70%-80%	70%-80%	70%-80%	50%-60%
CK	70%-80%	70%-80%	70%-80%	70%-80%	70%-80%

### 2.3 Measurements and calculations

#### 2.3.1 Photosynthetic indicators

Photosynthetic parameters were measured using an LI-6400 portable photosynthetic fluorometer (LI-COR, USA) from 09:00 to 11:00 on a typical sunny day after 5–7 d of water deficit treatment at all stages of watermelon fertility (seedling and maturity). Three watermelon plants were selected from each plot and measured alive in the field in the middle of the 5th fully expanded functional leaf from top to bottom. The net photosynthetic rate (*Pn*, μmol·m^-2^·s^-1^), transpiration rate (*Tr*, mmol·m^-2^·s^-1^), stomatal conductance (*Gs*, mmol·m^-2^·s^-1^), and intercellular CO_2_ concentration (*Ci*, μmol·mmol^-1^) were measured in 15–20 min to reduce time errors. Leaf water use efficiency (*WUEi = Pn/Tr*, μmol·mmol^-1^) and carboxylation rate (*CE = Pn/Ci*, mmol·m^-2^·s^-1^) were calculated according to the equations in parentheses.

#### 2.3.2 Fruit yield

Watermelons were harvested on August 19, 2020 and August 15, 2021, respectively, and the yield was measured by weighing the fruits marked in the plot, converted into the yield per hectare, with the mean value of three replicates taken as the actual fruit yield (t·ha^-1^) of each treatment.

#### 2.3.3 Fruit quality

The nutritional quality indicators of the selected watermelons were uniformly measured within one week following harvest, and the indicators and specific methods were determined as follows (Gao, 2000). Soluble solid (SS): by WAY-2S Abbe refractometer; Soluble protein (SP): by Komas Leuco G-250 staining; Vitamin C (Vc): by 2,6-dichloroindophenol titration; Organic acid (OA): by acid-base titration; Soluble sugar (SU): anthrone colorimetric method.

#### 2.3.4 Irrigation water use efficiency

The calculation formula for irrigation water use efficiency (t·ha^-1^·mm^-1^) is as follows:

(1) $$IWUE=\frac{Y}{I}$$

where Y is watermelon yield (t·ha^-1^); i is the amount of irrigation water (mm) for the whole growth stages of watermelon.

### 2.4 Multi-objective decision-making and evaluation based on a fuzzy matter-element model

The essence of the matter-element model (Wen, 1994) was used to promote the transformation of things and deal with incompatible problems, which is often used in multi-factor evaluation problems. On this basis, the concepts of fuzzy value and Euclidean proximity (distance) are introduced to rank the proximity of evaluation objects to standardized schemes based on multiple attributes. The specific analysis steps are as follows:

1. Construction of fuzzy matter-element (Wang and Xue, 2018)

Matter-element refers to the abbreviation of the ordered ternary R = (N, c, x) composed of the name (*N*) of the thing and the value (*x*) of its feature (*c*), and a thing is called a fuzzy matter-element when *x* is fuzzy. The *n*-dimensional matter-element combination of *m* things constitutes a compound element ( $R_{mn}$), where *n*-dimensional matter-element refers to the fact that *N* has *n* features and corresponding values. If the value of each feature in $R_{mn}$ is converted into a fuzzy value, it is called a compound fuzzy matter-element ( ${\underline{R}}_{mn}$).

(2) $$R_{mn}=[\begin{array}{cc} \begin{array}{cc} N_{1}\\ x_{1} & x_{11} \end{array} & \begin{array}{ccc} N_{2} & \cdots & N_{m}\\ x_{21} & \cdots & x_{m1} \end{array}\\ \begin{array}{cc} x_{2} & x_{12}\\ \vdots & \vdots \\ x_{n} & x_{1n} \end{array} & \begin{array}{ccc} x_{21} & \cdots & x_{m2}\\ \vdots & \ddots & \vdots \\ x_{2n} & \cdots & x_{mn} \end{array} \end{array}]$$

(3) $${\underline{R}}_{mn}=[\begin{array}{cc} \begin{array}{cc} N_{1}\\ x_{1} & u_{11} \end{array} & \begin{array}{ccc} N_{2} & \cdots & N_{m}\\ u_{21} & \cdots & u_{m1} \end{array}\\ \begin{array}{cc} x_{2} & u_{12}\\ \vdots & \vdots \\ u_{n} & u_{1n} \end{array} & \begin{array}{ccc} u_{21} & \cdots & u_{m2}\\ \vdots & \ddots & \vdots \\ u_{2n} & \cdots & u_{mn} \end{array} \end{array}]$$

Each evaluation factor has a corresponding fuzzy value, and the membership degree of the fuzzy value corresponding to each evaluation factor of the standard scheme is called the subordinate membership degree. The fuzzy value calculation equations for general different types of evaluation factors are:

(4) $$\text{Benefit type}:u_{ik}=x_{ik}/maxx_{ik}$$

(5) $$\text{Cost type}:u_{ik}=minx_{ik}/x_{ik}$$

where *x_ik_* and *u_ik_* are the quantitative and fuzzy values of the *k*-th feature (factor) of the *i*-th thing (scheme), respectively; max*x_ik_* and min*x_ik_* are the maximum and minimum of all quantitative values of each feature *x_ik_* in each thing, respectively.

2. Construction of difference square composite fuzzy matter element

The n-dimensional standard fuzzy matter-element ( $R_{0n}$) is composed of the maximum or minimum values extracted from the subordinate membership degree of each scheme and the square value ( $\Delta_{ij}$) of the difference between $R_{0n}$ and ${\underline{R}}_{mn}$ constitutes the difference-squared compound fuzzy matter-element matrix ( $R_{\Delta }$), i.e.:

(6) $$R_{\Delta }=[\begin{array}{cc} \begin{array}{cc} N_{1}\\ x_{1} & \Delta_{11} \end{array} & \begin{array}{ccc} N_{2} & \cdots & N_{m}\\ \Delta_{21} & \cdots & \Delta_{m1} \end{array}\\ \begin{array}{cc} x_{2} & \Delta_{12}\\ \vdots & \vdots \\ x_{n} & \Delta_{1n} \end{array} & \begin{array}{ccc} \Delta_{21} & \cdots & \Delta_{m2}\\ \vdots & \ddots & \vdots \\ \Delta_{2n} & \cdots & \Delta_{mn} \end{array} \end{array}]$$

3. The entropy weight method (EWM) is used to determine the weights of the participating indicators. For detailed calculation steps, please refer to the literature (Liu et al., 2021).

4. Calculation of Euclidean closeness and comprehensive score

Using ‘M (·, +)’ algorithm, then

(7) $$\;\rho H_{j}=1-\sqrt{\sum_{i=1}^{n}w_{i}^{*}\cdot \Delta_{ij}},\;\;\;j=1,\;2,\;\cdots ,\;m$$

(8) $$S=\rho H_{j}/\sum_{j=1}^{m}\rho H_{j}$$

where $\rho H_{j}$ is the mutual closeness between the *j*-th scheme and the standard scheme; *S* is the normalized Euclidean closeness, and $w_{i}^{*}$ is the weight of the feature (participation factor).

### 2.5 Data processing

The average and standard deviation (± SD) of the data were calculated in Microsoft Excel 2019 (Microsoft Corp., Raymond, Washington, USA). The software Origin 2020 (Origin Lab, Corp., Hampton, Massachusetts, USA) was used for graphing and regression analysis. Significance analysis with Duncan’s multiple range test was performed at a 5% level using SPSS 19.0 (IBM, Inc., New York, USA).

## 3 Results

### 3.1 Photosynthetic characteristics

**Figure 3:**
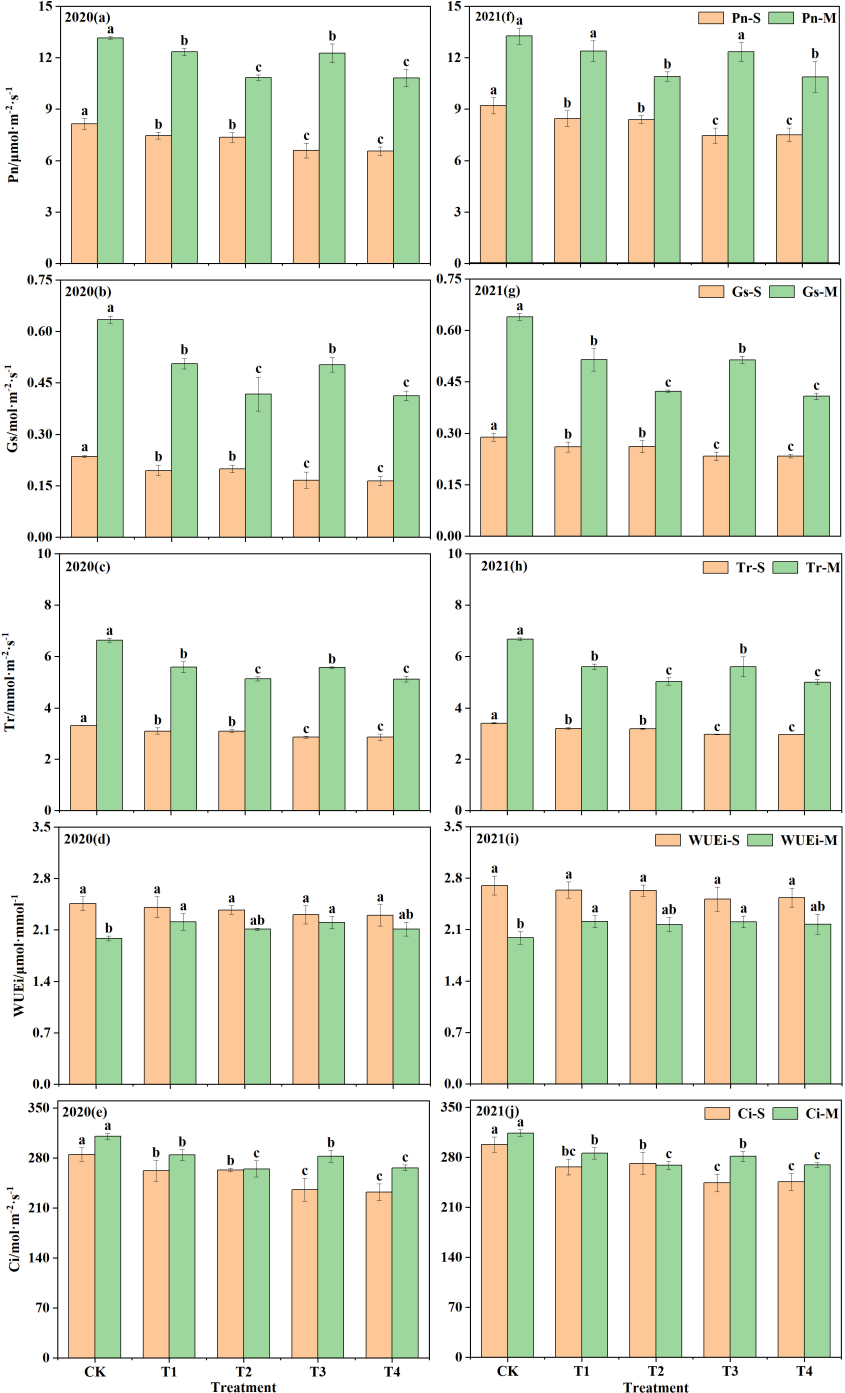
Effect of different water treatments on photosynthetic characteristics of watermelon in 2020 and 2021. "S" and "M" denote the seedling stage and the mature stage of the watermelon. T1, mild water deficit at both seedling and maturity stages; T2, mild water deficit at seedling stage and moderate water deficit at maturity stage; T3, moderate water deficit at seedling stage and mild water deficit at maturity stage; T4, moderate water deficit at both seedling and maturity stages; CK, adequate water supply throughout the reproductive period; Pn, net photosynthetic rate. Gs, stomatal conductance. Tr, transpiration rate. WUEi, leaf water use efficiency. Ci, intercellular CO2 concentration. Different lowercase letters represent significant differences at the P<0.05 level according to Duncan's multiple test.

Water deficit (WD) treatments for watermelon at multi-stage with under-film drip irrigation had significant effects on *Pn*, *Gs*, and *Tr* (**Figure 3**). For leaf *Pn*, moderate WD treatments at seedling stage (T3 and T4) decreased significantly (*P<*0.05) by 19.07–19.52% (2020) and 18.62–19.01% (2021) compared with CK, while mild WD treatments (T1 and T2) decreased at relatively low rates of 8.42–9.75% (2020) and 8.17–8.82% (2021), but the differences were also significant; Mild WD treatments at maturity (T1 and T3) decreased by 6.22–6.71% (2020) and 6.62–6.97% (2021) compared with CK, where the difference was not significant in 2021 (*P>*0.05), while moderate WD treatments (T2 and T4) decreased by 17.62–17.77% (2020) and 17.76–17.93% (2021), both of which were significantly different. As seen from leaf *Tr*, the T1 and T2 decreased by 6.37–6.44% (2020) and 6.11–6.44% (2021) compared with CK at the seedling stage, and both differences were significant, while the T3 and T4 treatments decreased significantly by 13.79–13.87% (2020) and 13.09–13.36% (2021); during the maturity stage, the T1 and T3 showed average decreases of 15.84% (2020) and 16.04% (2021), and T2 and T4 showed average decreases of 22.64% (2020) and 24.82% (2021), respectively, compared with CK, and all differences were significant. As shown by leaf *Gs*, compared with CK, both T3 and T4 reduced leaf *Gs* significantly at the seedling stage, with decreases ranging from 15.21–17.22% (2020) and 9.59–9.84% (2021), while T1 and T2 were also significantly lower than CK, with decreases ranging from 29.26–30.14% (2020) and 19.16–19.40% (2021); during the maturity stage, the T1 and T3 treatments decreased by 20.36–20.75% (2020) and 19.56–19.63% (2021) compared with CK, and the T2 and T4 both decreased by more than 30%, and all of them were significantly different. This indicates that WD could significantly reduce leaf *Pn*, *Tr*, and *Gs* at the beginning and end of the watermelon reproductive period, and all the decreases increase with the degree of WD.

WD significantly affected WUEi and *Ci* at the seedling and mature stages of watermelon. For leaf *Ci*, there were decreases in both the mild (T1 and T2) and moderate (T3 and T4) WD treatments at seedling stage compared with CK, with decreases of 7.66–8.01% and 17.37–18.51% in 2020 and 8.94–10.54% and 17.52–18.05% in 2021, respectively, all of which were significant (*P<*0.05) differences; Moderate WD treatments at maturity (T2 and T4) showed average decreases of 14.43% (2020) and 14.30% (2021) compared with CK, while mild WD treatments (T1 and T3) indicated average decreases of 8.66% (2020) and 9.74% (2021), both also significant differences. In terms of leaf WUEi, WD treatments at the seedling stage were all lower than CK, with decreases ranging from 2.04% to 6.78%, but were not significantly different from CK (*P>*0.05), and moderate WD (T3 and T4) were also lower than mild WD (T1 and T2); WD treatments at maturity were all higher than CK, among which mild WD treatments (T1 and T3) were significantly higher than CK, with increases of 10.94–11.44% (2020) and 10.99–11.11% (2021), while moderate WD treatments (T2 and T4) were also higher than CK, but both were at the same level. This demonstrates that WD significantly inhibits transpiration by increasing leaf stomatal resistance, but is relatively insensitive to the effect on *Pn*.

### 3.2 Fruit yield and irrigation water use efficiency

**Figure 4:**
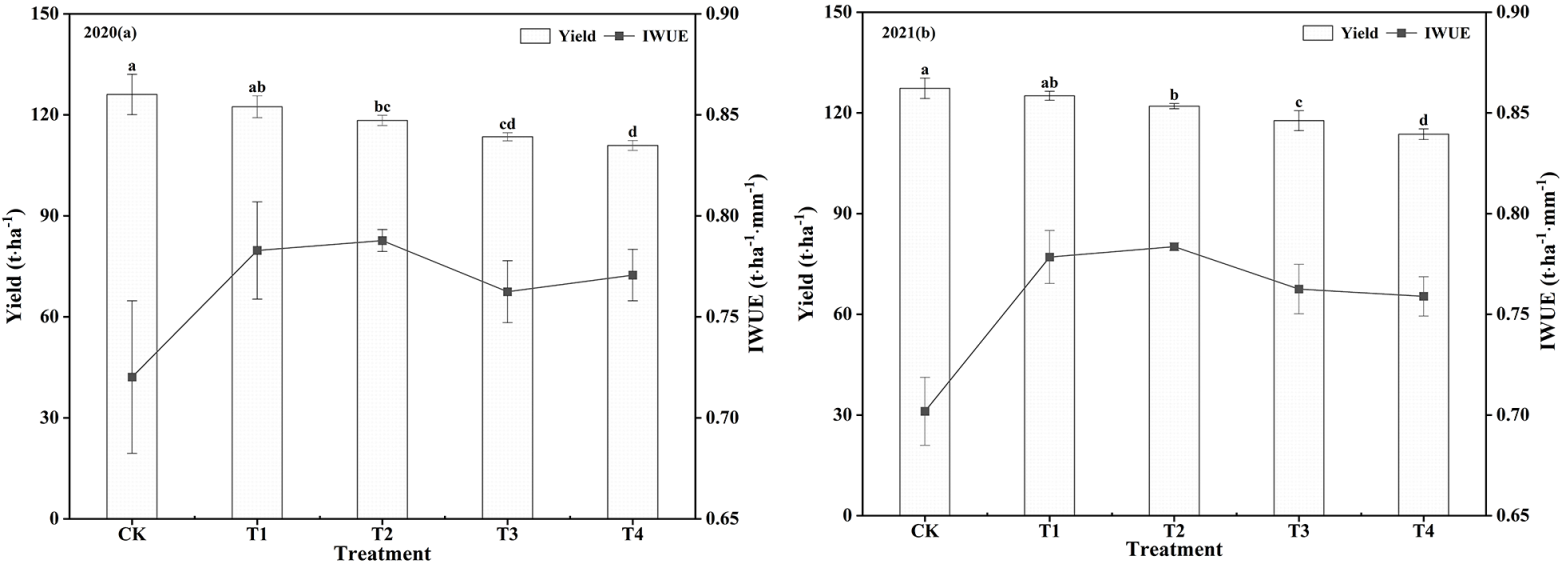
Effect of different water treatments on watermelon fruit yield and IWUE in 2020 and 2021. T1, mild water deficit at both seedling and maturity stages; T2, mild water deficit at seedling stage and moderate water deficit at maturity stage; T3, moderate water deficit at seedling stage and mild water deficit at maturity stage; T4, moderate water deficit at both seedling and maturity stages; CK, adequate water supply throughout the reproductive period; IWUE, irrigation water use efficiency. Different lowercase letters represent significant differences at the P<0.05 level according to Duncan's multiple test.

Under-film drip irrigation regulated deficit significantly affected watermelon fruit yield in both years (**Figure 4**). The fruit yield of CK was the highest, while the yield of multi-stage WD treatment was lower than that of CK. The fruit yield of T1 treatment was not significantly different from that of CK (*P>*0.05), which was 122.40 t·ha^-1^ in 2020 and 125.08 t·ha^-1^ in 2021, but the other treatments were significantly lower than CK (*P<*0.05) by 6.10–11.99% (2020) and 4.17–10.74% (2021). WD had a significant effect on irrigation water use efficiency (IWUE) (**Figure 4**). The IWUE of CK was the lowest, while the T2 treatment had the highest IWUE with a significant increase of 9.38% (2020) and 11.66% (2021) compared with CK. The IWUE of the remaining treatments decreased by 5.87–8.70% (2020) and 8.13–10.92% (2021) compared with CK, and all differences were significant. Moreover, the yield and IWUE of T1 were greater than those of T4, while the yield and IWUE of T3 were less than those of T2, indicating that watermelon yield decreased with increasing WD, and the effect of WD at the seedling stage on yield was greater than at the maturity stage.

### 3.3 Fruit quality

Compared with CK, multi-stage WD significantly (*P<*0.05) affected fruit nutritional quality indicators (**Table 2**). The fruit SS, SU, VC, and SP contents increased by 8.69–12.50%, 17.36–32.41%, 15.95–23.64%, and 7.02–10.11% in 2020, and 8.31–12.51%, 17.38–32.49%, 14.68–22.09% and 6.92–11.51%; while OA content, except for moderate WD at the maturity stage in 2021, was not significantly different from CK in all WD treatments (*P>*0.05). Besides, among the combinations of WD at seedling and maturity stages, the quality indicator values of moderate WD at the maturity stage were significantly higher than those of mild WD, with T2 >T1 and T4 >T3, and in which both SU and SP contents were significantly different; Furthermore, T4 > T1 under the same WD level, while T2 > T3 under different WD levels at the same period. This revealed that WD at the maturity stage had a greater effect on fruit quality and that quality improved significantly with increasing WD.

**Table 2:** Effect of different water treatments on the nutritional quality of watermelon in 2020 and 2021.

Year	Treatments	SS/%	OA/%	SU/%	Vc/mg·100g^-1^	SP/mg·100g^-1^	SSOA	SUOA
2020	T1	12.71 ± 0.39a	0.19 ± 0.02a	9.66 ± 0.11b	9.27 ± 0.45a	41.49 ± 0.58b	66.85 ± 6.55a	50.78 ± 4.10a
	T2	13.12 ± 0.72a	0.20 ± 0.01a	10.90 ± 0.17a	9.79 ± 0.94a	42.61 ± 0.42a	64.25 ± 5.40a	53.31 ± 1.36a
	T3	12.68 ± 0.51a	0.19 ± 0.01a	9.87 ± 0.10b	9.27 ± 0.34a	41.49 ± 0.75b	65.56 ± 0.52a	51.10 ± 2.09a
	T4	12.98 ± 0.40a	0.21 ± 0.02a	10.90 ± 0.08a	9.88 ± 0.82a	42.68 ± 0.53a	63.64 ± 6.92a	53.38 ± 4.49a
	CK	11.66 ± 0.44b	0.18 ± 0.01a	8.23 ± 0.40c	7.99 ± 0.06b	38.77 ± 0.12c	63.18 ± 2.27a	44.58 ± 1.67b
2021	T1	12.73 ± 0.60a	0.19 ± 0.00b	9.65 ± 0.10b	9.31 ± 0.39ab	41.34 ± 0.33b	66.59 ± 2.49a	50.52 ± 0.83a
	T2	13.15 ± 0.50a	0.21 ± 0.00a	10.89 ± 0.19a	9.79 ± 0.35a	43.12 ± 0.46a	63.53 ± 3.06a	52.62 ± 1.30a
	T3	12.66 ± 0.34a	0.19 ± 0.01b	9.86 ± 0.10b	9.21 ± 0.11b	41.50 ± 0.20b	65.55 ± 1.06a	51.06 ± 1.67a
	T4	13.13 ± 0.29a	0.21 ± 0.01a	10.89 ± 0.08a	9.80 ± 0.19a	42.65 ± 0.50a	63.66 ± 2.60a	52.82 ± 2.36a
	CK	11.69 ± 0.50b	0.19 ± 0.01b	8.22 ± 0.40c	8.03 ± 0.21c	38.67 ± 0.11c	62.57 ± 4.70a	44.04 ± 3.84b

T1, mild water deficit at both seedling and maturity stages; T2, mild water deficit at seedling stage and moderate water deficit at maturity stage; T3, moderate water deficit at seedling stage and mild water deficit at maturity stage; T4, moderate water deficit at both seedling and maturity stages; CK, adequate water supply throughout the reproductive period; SS, soluble solids; OA, organic acids; SU, soluble sugars; Vc, vitamin C; SP, soluble protein; SSOA, solid to acid ratio; SUOA, sugar to acid ratio. Different lowercase letters represent significant differences at the *P* < 0.05 level according to Duncan’s multiple test.

### 3.4 Correlation analysis and multi-objective decision-making based on fuzzy matter-element model

**Figure 5:**
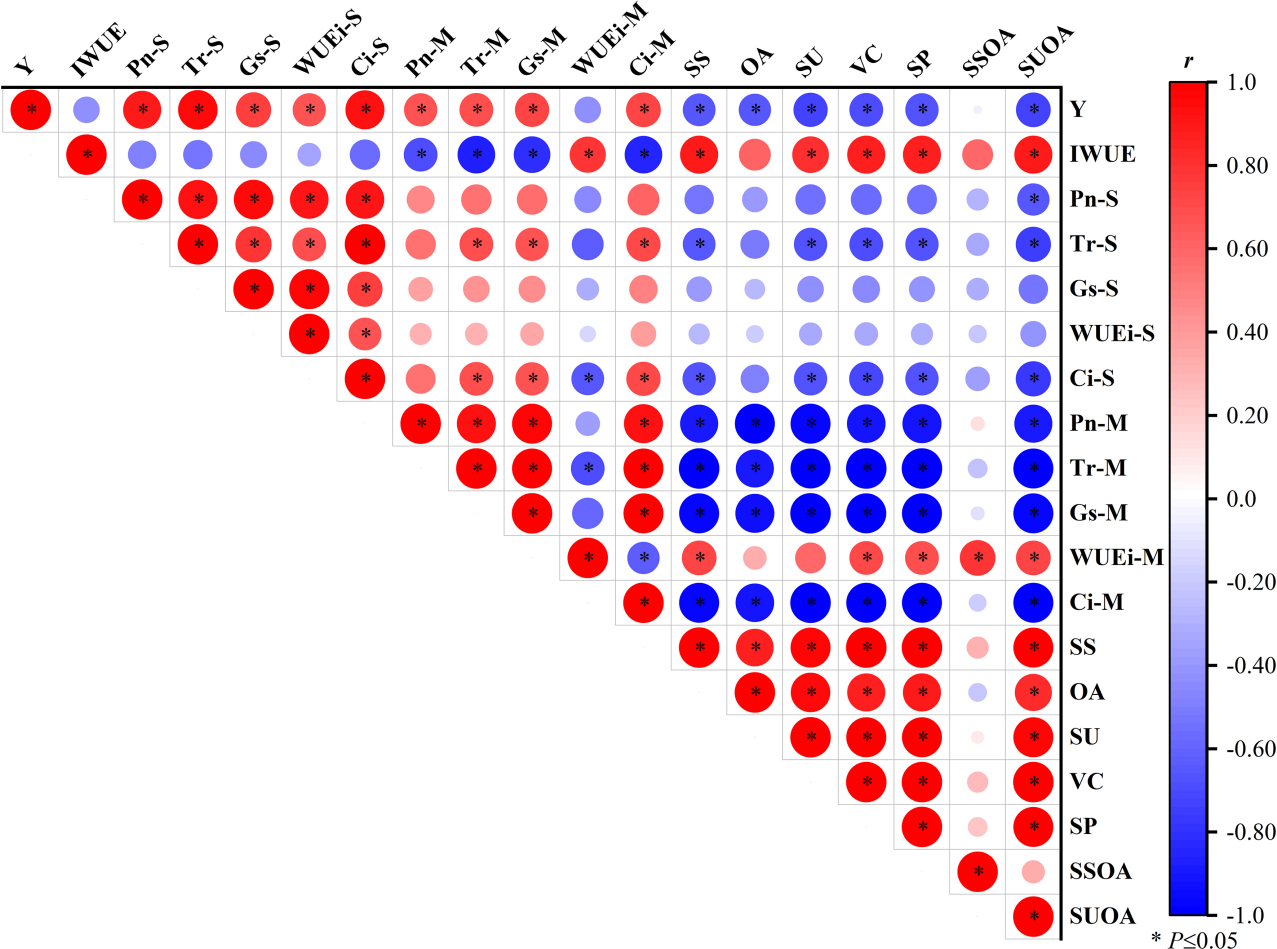
Correlation analysis of watermelon fruit yield, irrigation water use efficiency, photosynthetic characteristics, and quality indicators based on the mean values of 2020 and 2021. Y, watermelon fruit yield; IWUE, irrigation water use efficiency; Pn-S, net photosynthetic rate at seedling stage; Tr-S, transpiration rate at seedling stage; Gs-S, stomatal conductance at seedling stage; WUEi-S, leaf water use efficiency at seedling stage; Ci-S, intercellular CO2 concentration at seedling stage; Pn-M, net photosynthetic rate at maturity stage; Tr-M, transpiration rate at maturity stage; Gs-M, stomatal conductance at maturity stage; WUEi-M, leaf water use efficiency at maturity stage; Ci-M, intercellular CO2 concentration at maturity stage; SS, soluble solids; OA, organic acids; SU, soluble sugars; Vc, vitamin C; SP, soluble protein; SSOA, solid to acid ratio; SUOA, sugar to acid ratio.

As shown in **Figure 5**, watermelon yield was significantly (*P<*0.05) correlated with *Pn* (0.886, 0.669), *Tr* (0.954, 0.696), *Gs* (0.746, 0.721), and *Ci* (0.930, 0.728) at the seedling and maturity stages; IWUE was also significantly correlated with WUEi of the maturity stage (0.797); There were significant correlations between SS and WUEi of the maturity stage (0.739), Vc and WUEi of the maturity stage (0.703), and SP and WUEi of the maturity stage (0.699), while the correlation coefficients of OA and SU with WUEi of the maturity stage were 0.335 and 0.589 respectively, which did not reach a significant level. It can be seen that the relationship between watermelon fruit yield and photosynthetic characteristics at the seedling stage is relatively closer than that at the maturity stage, but fruit quality is more closely related to photosynthetic characteristics at the maturity stage than that at the seedling stage.

With watermelon fruit yield, nutrient quality, and IWUE (Equation 1) as evaluation indicators, the Euclidean closeness of 2a calculated based on the fuzzy matter-element model (Equations 2-6) and its normalized ranking are shown in **Table 3**. The Euclidean closeness (ρH) of each treatment was calculated separately from (Equation 7) using the weighting coefficients derived from the difference-squared compound fuzzy element (RΔ) and the entropy weighting method, and then normalized and ranked by (Equation 8).

**Table 3:** Analysis of watermelon yield, quality, and IWUE based on weighted fuzzy matter-element model.

Year	Treatments	Weighted difference-square compound fuzzy matter-element(×10^-3^)							*ρH*	*S*	Ranking
		Y	SS	OA	SU	Vc	SP	IWUE			
2020	T1	0.130	0.128	0.501	1.803	0.492	0.102	0.000	0.871369	0.187913	5
	T2	0.590	0.000	1.807	0.000	0.013	0.000	0.000	0.943813	0.203536	2
	T3	1.575	0.147	0.501	1.268	0.509	0.104	0.089	0.950898	0.205064	1
	T4	2.283	0.016	1.807	0.000	0.000	0.000	0.022	0.935246	0.201689	4
	CK	0.000	1.550	0.000	8.360	4.726	1.110	0.801	0.935738	0.201795	3
2021	T1	0.045	0.144	0.510	1.839	0.339	0.229	0.022	0.867517	0.186799	5
	T2	0.257	0.000	1.840	0.000	0.000	0.000	0.000	0.944071	0.203283	2
	T3	0.850	0.194	0.510	1.294	0.508	0.192	0.089	0.954198	0.205464	1
	T4	1.709	0.001	1.840	0.000	0.000	0.016	0.201	0.939691	0.202340	3
	CK	0.000	1.679	0.000	8.527	4.474	1.445	1.427	0.938626	0.202111	4

T1, mild water deficit at both seedling and maturity stages; T2, mild water deficit at seedling stage and moderate water deficit at maturity stage; T3, moderate water deficit at seedling stage and mild water deficit at maturity stage; T4, moderate water deficit at both seedling and maturity stages; CK, adequate water supply throughout the reproductive period; Y, watermelon fruit yield; SS, soluble solids; OA, organic acids; SU, soluble sugars; Vc, vitamin C; SP, soluble protein; IWUE, irrigation water use efficiency; *ρH*, Euclidean closeness; *S*, normalized score.

According to the normalized Euclidean proximity ranking results, the ranking of the five water management modes for 2 years was basically the same, from best to worst, T2 >T1 > T4 ≈ T3 > CK. Hence, we concluded that the optimal multi-stage deficit irrigation mode for watermelon under film drip irrigation in arid oasis region is T2 based on fuzzy matter-element model, i.e., the mild (60–70% FC) deficit at the seedling stage and moderate (50–60% FC) deficit at the maturity stage, while T1 treatment, i.e., mild (60–70% FC) deficit at both seedling and maturity stage can be considered as an alternate mode.

## 4 Discussion

### 4.1 Effects of WD on photosynthetic characteristics

Photosynthetic assimilation is the most critical life activity of plants, which is largely affected by the plant itself and external environmental factors, and is relatively sensitive to abiotic stress. Water is a common factor that can significantly affect plant growth and physiological and biochemical processes (Ashraf and Harris, 2013; Nadal and Flexas, 2019; Kumar et al., 2023). Our results showed that *Pn*, *Gs*, *Tr*, and *Ci* of WD treatment at the seedling stage or maturity stage were significantly lower than those of CK. This may be due to the signal of drought stress in the root system being transmitted to the leaves through related pathways, causing the decrease of stomatal opening on the leaf surface, reducing CO_2_ uptake, increasing the resistance of mesophyll cells, reducing the activity of related enzymes in the process of photosynthesis, and ultimately affecting the fixation and reduction of CO_2_, thereby resulting in a reduction in the *Pn* and *Tr* of leaves (Osakabe et al., 2014; Fila et al., 2019). This phenomenon has been observed in numerous species, including herbaceous crops such as tobacco (Franco-Navarro et al., 2021), tomato (Gaion and Carvalho, 2021), common bean (Rosales et al., 2013), and maize (Vennam et al., 2023), as well as tree species like citrus (Miranda et al., 2022) and apple (Yang et al., 2023), and is further supported by the findings of Akhoundnejad and Dasgan (2020) in drought-stressed melon. The study also revealed that the WUEi of the mild WD treatment at the seedling stage was not significantly different from that of CK, but that the mild WD treatment at the maturity stage was significantly higher than CK. This is probably due to the smaller decrease in *Pn* and a larger decrease in *Tr* with the mild WD treatment compared with other treatments. This is consistent with the findings of (Yang et al., 2023) and Li et al. (2022) for apple and tomato, respectively. However, it has also been shown that WD leads to a decrease in *Gs* and *Tr*, thereby reducing *Pn*, and a decrease in WUEi (Zheng et al., 2013). This phenomenon may be related to the degree of WD and the species of crop.

### 4.2 Effects of WD on fruit yield, quality, and IWUE

Water is the most important environmental limiting factor in agricultural production (Wu et al., 2022). But WD does not always reduce crop yield, and appropriate WD treatment can improve product quality under the premise of stable and even increased yield (Yang et al., 2018; Ozer et al., 2020). In this study, watermelon yields in the T1 were only 1.75%–2.87% lower than CK, while the remaining treatments significantly reduced yields by 4.17%–11.99%, with non-significant yield differences between the T1 and T2. The reason for this result may be that the suppressive effect of heavy water stress on the above-ground organs of the plant at the beginning of the reproductive period (seedling stage) was not fully compensated for by the re-watering at a later stage (Wang et al., 2020a); moreover, the WD treatment at the maturity stage would, to some extent, lead to a decrease in the water content of the fruit, and thus a decrease in yield, especially when the WD was large. These results are similar to those of Cui et al. (2020) for WD tomato, but the opposite results were obtained by Cui et al. (2008) for pear dates. The results of this study also showed that multi-stage WD treatments significantly increased the IWUE of watermelon. The deeper reason for this may be that water demand is relatively low at the beginning and end of the watermelon reproductive period, and applying water regulation at this stage not only saves irrigation but also has less impact on yield per plant than that of WD treatments at the expansion stage (Abdelkhalik et al., 2019). Similar findings have been reported in prior studies by Zhang et al. (2023). Furthermore, the SS, SU, Vc, and SP contents of fruits treated with WD at multi-stages were increased, especially in the moderate WD treatment at the maturity stage. This may be because WD treatment at the maturity stage reduces the water potential of fruit cells and enhance the ability of cells to absorb water and nutrients from the outside world, thus effectively improving the nutritional quality of fruits (Liu et al., 2021). This is similar to the conclusion of Kuscu et al. (2015). Meanwhile, the application of water stress during the maturation stage promotes starch accumulation in immature fruits, thereby enhancing the synthesis and translocation of photosynthetic products in the form of sucrose to reproductive organs, which subsequently increases the content of soluble sugar (SS) in fruits (Liu et al., 2019; Sun et al., 2023). A positive correlation is observed between soluble sugar content and vitamin C content (**Figure 5**). Since sugars serve as key precursors for vitamin C biosynthesis, the water stress-induced enhancement of starch accumulation and its conversion into sugars contributes to the increased levels of vitamin C (Boverio et al., 2024). He et al. (2024) reported similar findings in their study on tomatoes.

### 4.3 Integrated evaluation of fuzzy matter-element model based on entropy weight method

In this study, a fuzzy matter-element model was used to comprehensively evaluate the yield, quality, and IWUE of five multi-stage deficit irrigation modes. The comprehensive evaluation method of the watermelon deficit irrigation effect based on coupling physical element analysis theory and fuzzy quantitative values overcomes the problem of the limited reliability of a single evaluation model, and the entropy weight method determines the weights of each evaluation indicator, thereby enhancing the rationality of water-saving irrigation scheme optimization for watermelon under film drip irrigation. Although the comprehensive evaluation results of the entropy-fuzzy matter-element model of 2a are slightly different, the overall benefit of T2 is the best, followed by T1. Thus, multi-stage treatment of watermelon with mild WD at the seedling stage and moderate or mild WD at the maturity stage could improve IWUE and fruit nutritional quality under the premise of stable yield. This method also provides a practical reference for the analysis and evaluation of irrigation effects in similar crops.

## 5 Conclusion

In the arid oasis area, optimizing water management is crucial for achieving water-saving and high-quality watermelon production. Appropriate water regulation at different growth stages can significantly affect the photosynthetic characteristics, yield formation, quality composition, and water use efficiency of watermelons. The results of this study show that compared with the full-irrigation treatment throughout the growth period (CK), implementing different levels of water deficit combinations during the seedling and mature stages of watermelon reduces the *Pn*, *Gs*, *Tr*, and *Ci* of watermelon leaves, but increases WUEi. Within the same period, *Pn*, *Gs*, *Tr*, and *Ci* of watermelon decrease with the intensification of water deficit. Compared with CK, T1 treatment can ensure watermelon yield, increase IWUE, and increase the SS, SU, Vc, and SP content of the fruit; while the yield of T2 treatment is lower than CK, its irrigation water use efficiency is the highest and the nutritional quality of the fruit is also significantly improved. The comprehensive evaluation based on the entropy weight-fuzzy matter-element model indicates that T2 treatment has the best overall performance, followed by T1. Therefore, the two-stage irrigation mode of mild water deficit during the seedling stage combined with moderate (T2) or mild water (T1) deficit during the mature stage is a relatively suitable water-saving irrigation strategy in the arid area of the Hexi Corridor. It can significantly improve water use efficiency and fruit quality while ensuring stable yield, providing a scientific basis for efficient water use and high-quality cultivation of watermelons in this region.

## Data Availability

The original contributions presented in the study are included in the article/supplementary material. Further inquiries can be directed to the corresponding author.
